# Supplementary material for: Genetic Variants of Glucose-6-Phosphate Dehydrogenase and Their Associated Enzyme Activity: A Systematic Review and Meta-Analysis
Source: Pathogens. 2022 Sep 14;11(9):1045. doi: 10.3390/pathogens11091045 (PMC9502867; doi:10.3390/pathogens11091045)
Supplement: Supplementary file 1 [file pathogens-11-01045-s001.zip › Supplementary File S1.pdf]

Supplementary File S1: Literature search protocols

Search terms:

(G6PD OR Glucose-6-Phosphate Dehydrogenase OR “Glucose 6 Phosphate Dehydrogenase”) AND (spectrophotometry OR quant* OR Activity) AND (genotype OR geno* OR variant)

Inclusion and exclusion criteria:

Study Criteria

Inclusion:

- Able to provide individual-level data for phenotype and genotype
- Used one of the following quantitative spectrophotometry assays: Trinity, Randox, Pointe Scientific
- Performed G6PD genotyping (either full sequencing or SNP-typing)

Exclusion:

- Studies published before 1983
- Studies with <30 males
- Studies published in a language other than English

Participant Criteria:

Inclusion:

- All ages
- Males and females
- Malaria positive and malaria negative

Exclusion:

- Blood transfusion in the last 4 months (if known) –
- Any other event that, may have caused considerable blood loss (injury, birth, if known…)
- Other known haematological conditions (e.g. thalassemia, sickle cell …)

Data variables collected

Essential*:*

- Phenotypic method
- Molecular method
- Phenotypic G6PD result
- G6PD variant
- Hb
- Sex
- Malaria status in malaria endemic areas
- Age

Desirable:

- Multiple measurements that contributed to the final phenotypic G6PD result
- Body temp
- History of febrile disease in the last 6 months (malaria, dengue etc)
- History of blood transfusion in the last 6 months
- History of injury & pregnancy/birth
