# Supplementary material for: Genetic Variants of Glucose-6-Phosphate Dehydrogenase and Their Associated Enzyme Activity: A Systematic Review and Meta-Analysis
Source: Pathogens. 2022 Sep 14;11(9):1045. doi: 10.3390/pathogens11091045 (PMC9502867; doi:10.3390/pathogens11091045)
Supplement: Supplementary file 1 [file pathogens-11-01045-s001.zip › Supplementary File S2.pdf]

Supplementary File S2: Modified QUADAS tool

**Domain 1: Patient selection**	
**A. Risk of bias**	
**Describe methods of patient selection:**	
• **Was a consecutive or random sample of patients enrolled?**	Yes/No/Unclear
• **Did the study avoid inappropriate exclusions?**	Yes/No/Unclear
**Could the selection of patients have introduced bias?**	RISK: LOW/HIGH/UNCLEAR
**B. Concerns regarding applicability**	
**Describe criteria for genotyping**	
**Is there concern that the included patients do not match the review question?**	CONCERN: LOW/HIGH/UNCLEAR
**Domain 2: DETERMINING GENOTYPE** ***(if more than 1 index test was used, please complete for each test)***	
**A. Risk of bias**	
**Describe how genotype was identified**	
• **Was genotype determined without knowledge of G6PD activity?**	Yes/No/Unclear
**Could the conduct or interpretation of the index test have introduced bias?**	RISK: LOW/HIGH/UNCLEAR
**Domain 3: DETERMINING PHENOTYPE**	
**A. Risk of bias**	
**Describe the reference standard and how it was conducted and interpreted:**	

• **Is the reference standard likely to correctly classify the target condition?**	Yes/No/Unclear
• **Were the reference standard results interpreted without knowledge of the results of the index test?**	Yes/No/Unclear
**Could the reference standard, its conduct, or its interpretation have introduced bias?**	RISK: LOW/HIGH/UNCLEAR
**Domain 4: Flow and timing**	
**A. Risk of bias**	
• **Was there an appropriate interval between sample collectionand spectrophotometry (<7days at 4°C)?**	Yes/No/Unclear
• **Did patients receive the same variuant determiantion and spectrophotometry ?**	Yes/No/Unclear
• **Were all patients included in the analysis?**	Yes/No/Unclear
**Could the patient flow have introduced bias?**	RISK: LOW/HIGH/UNCLEAR
