# Supplementary material for: Genetic Variants of Glucose-6-Phosphate Dehydrogenase and Their Associated Enzyme Activity: A Systematic Review and Meta-Analysis
Source: Pathogens. 2022 Sep 14;11(9):1045. doi: 10.3390/pathogens11091045 (PMC9502867; doi:10.3390/pathogens11091045)
Supplement: Supplementary file 1 [file pathogens-11-01045-s001.zip › Supplementary File S3.pdf]

Supplementary File S3: QUADAS Results

Domain 1: Patient Selection

Study	Recruitment	Enrolment consecutive /random?	Exclusions avoided?	Did selection introduce bias?	Criteria for genotyping	Risk of bias from patients matching review Q?
**Bancone_et_al_ 2015 (27)**	Purposive selection of known G6PD phenotype or genotype	no	yes	high	All participants	low
**Bancone_et_al_ 2016 (28)**	Exhaustive cross-sectional surveys	yes	yes	low	Phenotypically deficient males, all females	low
**Bancone_et_al_ 2017 (29)**	Health-facility based recruitment, pregnant women attending antenatal care	yes	yes	low	Genotyping for Mahidol was performed on all included women, while Chinese-4, Kaiping, Canton and Mediterranean variants were analysed only on women with either enzymatic activity below 80% of normal in Mahidol-wild type genotype or enzymatic activity below 30% and Mahidol-heterozygous genotype.	unclear
**Brito_et_al_2016 (30)**	Consecutive males, malaria patients from clinic, non-malaria from clinic and community	yes	no	high	Only genotyped those with G6PD <70%, 100% based on a mean of males, not median	low
**Deng_et_al_2017 (31)**	Unclear, 100 unrelated individuals fitting inclusion criteria were enrolled	no	unclear	high	All phenotypically deficient participants genotyped	low

**Johnson_et_al_ 2009 (32)**	random selection post census, only children 1-10 yrs, all participants genotyped, some standardized exclusion criteria	yes	no	low	110 randomly selected individuals tested for A-	low
**Kim_et_al_2011 (33)**	Cross sectional survey, two stage cluster sampling, 1 child / household	unclear	unclea r	unclear	All phenotypically deficient participants genotyped (<4.5U/gHb)	low
**La_Rue_et_al_2 014 (34)**	Blood samples from African-American blood donors	no	no	high	Genotyping selection not specified, but state it was biased towards lower G6PD activity	low
**Ley_et_al_2021 (35)**	Composite data from three studies: clinical trial of malaria patients, cross-sectional survey, case-control study	yes	yes	low	all individuals <70% AMM and a sample subsample above 70%	low
**Pal_et_al_2021 (36)**	Composite data from multiple studies: 3 diagnostic accuracy studies in USA, focusing on African-Americans in recruitment. 1 study in UK -requested testing already completed at Hammersmith Hospital Haematology laboratory	no	no	high	limited number', 'covering the critical G6PD activity dynamic range'	low
**Reading_et_al_ 2016 (37)**	Patients presenting with AHA + health controls, patients followed up 4+ months following AHA.	yes	yes	low	All AHA patients and all healthy controls	low

**Roca-Feltrer_et_al_2014 (38)**	Patients enrolled based on pre-defined eligibility criteria, how patients were identified is unclear	unclear	no	high	All individuals with <60% of AMM	low
**Roh_et_al_2016 (39)**	Cross sectional survey, two stage cluster sampling, 1 child / household	yes	no	low	625 / 631 genotyped	low
**Satyagraha_et_al_2015 (40)**	Cross-sectional surveys, villages in proximity to health centres, all residents invited to participate.	yes	yes	low	all G6PD deficient + convenience sample of G6PD normal. 80/104 agreed for genotyping.	low
**Satyagraha_et_al_2016 (41)**	Random selection of all residents of one village.	yes	yes	low	all G6PD deficient + those with low HB (<8g/dL)	low
**Satyagraha_et_al_2021 (42)**	Cross-sectional surveys, females >6yrs	unclear	yes	low	all individuals <80% normal activity	low
**Xia_et_al_2016 (43)**	Hospital-based sampling, method comparison study	unclear	yes	unclear	G6PD deficient by novel assay and/or G6PD/6PGD ratio + 80 randomly selected G6PD normal (40 each sex).	low

Domain 2: Genotype

Study	How was genotyping done?	Was genotyping done without knowing spec results?	Could genotyping have introduced bias?
**Bancone_et_al_2015 (27)**	Sequencing	no	high
**Bancone_et_al_2016 (28)**	PCR-RFLP for Mahidol, Chinese-4, Canton, Viangchan	no	high
**Bancone_et_al_2017 (29)**	PCR-RFLP for Mahidol, Chinese-4, Kaiping, Mediterranean, Canton	no	high
**Brito_et_al_2016 (30)**	PCR-RFLP for A-, Chatham, Mediterranean, Aures	no	high
**Deng_et_al_2017 (31)**	SNaPshot assay	no	high
**Johnson_et_al_2009 (32)**	Genotyping for 3 A- variants	unclear	unclear
**Kim_et_al_2011 (33)**	Sequencing	no	high

**La_Rue_et_al_2014 (34)**	Sequencing	no	high
**Ley_et_al_2021 (35)**	PCR-RFLP for Mahidol, Viangchan, Mediterranean, Orissa, Kalyan-Kerala	no	high
**Pal_et_al_2021 (36)**	Sequencing	no	high
**Reading_et_al_2016 (37)**	SNaPshot assay	yes	low
**Roca-Feltrer_et_al_2014 (38)**	Sequencing	no	high
**Roh_et_al_2016 (39)**	PCR-RFLP for A-	unclear	unclear
**Satyagraha_et_al_2015 (40)**	Sequencing	no	high
**Satyagraha_et_al_2016 (41)**	PCR-RFLP for Vanua Lava, Chatham, Viangchan and Kaiping	no	high
**Satyagraha_et_al_2021 (42)**	PCR-RFLP for Vanua Lava, Chatham, Viangchan, Coimbra, Kaiping + sequencing for indeterminate results	no	high
**Xia_et_al_2016 (43)**	Multicolour melting curve analysis	no	high

Domain 3: Phenotype

**Study**	**How was spec done?**	**Were replicate measures used?**	**Were control samples used regularly?**	**Was reaction temperature addressed?**	**Risk of bias due to spec. assay and/or procedures?**	**Was malaria status considered when defining deficiency?**	**Was spec done without knowing genotype?**	**Was '100% activity' defined based on study's own data?**	**Risk of bias due to spec methods and/or interpretation?**
**Bancone_et_al_2015 (27)**	Duplicate testing using trinity, AMM from 26 normal males	yes	yes	yes	low	yes	yes	yes	low

**Bancone_et_al_2016 (28)**	Trinity, unclear if replicates used. Previously established AMM of 7.51 U/gHb used	yes	yes	yes	low	no	yes	yes	low
**Bancone_et_al_2017 (29)**	WHO method on WBC depleted samples done in triplicates. As only pregnant women, 'female population median' was used, 11.5U/g Hb	yes	yes	yes	low	no	yes	yes	high
**Brito_et_al_2016 (30)**	Pointe Scientific	yes	yes	yes	low	yes	yes	yes	low
**Deng_et_al_2017 (31)**	Single testing with Trinity, deficiency defined as <4.5U/gHb	no	yes	yes	high	no	yes	unclear	high
**Johnson_et_al_2009 (32)**	Single testing with Randox, time to testing not mentioned, deficiency defined from Randox recommendation	no	yes	yes	high	no	unclear	no	high
**Kim_et_al_2011 (33)**	Single testing with Trinity, deficiency defined as <4.5U/gHb, 100% is a mean, not an AMM	no	yes	yes	high	no	yes	yes	high
**La_Rue_et_al_2014 (34)**	Trinity, AMM of7.18	yes	yes	yes	low		yes	yes	low
**Ley_et_al_2021 (35)**	Randox + Pointe Scientific	yes	yes	yes	low	yes	yes	yes	low

**Pal_et_al_2021 (36)**	Pointe Scientific	no	yes	yes	high		yes	unclear	unclear
**Reading_et_al_2016 (37)**	Randox	no	yes	yes	low	no	yes	no	low
**Roca-Feltrer_et_al_2014 (38)**	Single testing with Trinity, deficiency defined as <60%	no	yes	yes	high	no	yes	yes	high
**Roh_et_al_2016 (39)**	Single testing with Trinity done within 24 hours, deficiency defined as 60% of AMM	no	yes	yes	high	no	unclear	yes	high
**Satyagraha_et_al_2015 (40)**	Trinity	no	yes	yes	high	no	yes	no	high
**Satyagraha_et_al_2016 (41)**	Trinity, in triplicate	yes	yes	yes	low	no	yes	no	high
**Satyagraha_et_al_2021 (42)**	Trinity	no	yes	yes	high	yes	yes	no	high
**Xia_et_al_2016 (43)**	Zhongshan Biotech G6PD/6PGD ratio	unclear	yes	yes	unclear	no	yes	unclear	unclear

Domain 4: Flow and timing

Study	Was spec done within 7d (sample at 4°C)?	Did all patients receive the same testing?	Were all patients included in analysis?	Could patient flow have introduced bias?
**Bancone_et_al_2015 (27)**	yes	yes	yes	low
**Bancone_et_al_2016 (28)**	yes	yes	no	low

**Bancone_et_al_2017 (29)**	yes	yes	yes	low
**Brito_et_al_2016 (30)**	yes	yes	yes	high
**Deng_et_al_2017 (31)**	unclear	yes	no	high
**Johnson_et_al_2009 (32)**	unclear	yes	no	low
**Kim_et_al_2011 (33)**	yes	yes	no	high
**La_Rue_et_al_2014 (34)**	yes	yes	yes	low
**Ley_et_al_2021 (35)**	yes	no	yes	high
**Pal_et_al_2021 (36)**	yes	yes	yes	high
**Reading_et_al_2016 (37)**	unclear	no	no	high
**Roca-Feltrer_et_al_2014 (38)**	yes	yes	no	high
**Roh_et_al_2016 (39)**	yes	yes	no	low
**Satyagraha_et_al_2015 (40)**	yes	yes	no	high
**Satyagraha_et_al_2016 (41)**	yes	yes	no	high
**Satyagraha_et_al_2021 (42)**	yes	yes	no	high
**Xia_et_al_2016 (43)**	yes	yes	no	high
