# Supplementary material for: Genetic Variants of Glucose-6-Phosphate Dehydrogenase and Their Associated Enzyme Activity: A Systematic Review and Meta-Analysis
Source: Pathogens. 2022 Sep 14;11(9):1045. doi: 10.3390/pathogens11091045 (PMC9502867; doi:10.3390/pathogens11091045)
Supplement: Supplementary file 1 [file pathogens-11-01045-s001.zip › Supplementary Table S1.pdf]

Table S1: Characteristics of Included Studies

Study	Country	Study format	Sample Size	Number genotyped	Spectro. assay	Genotyping method	AMM (U/g Hb)	Sex (% Male*)	Age in years (mean (range))	Malaria (% Positive*)
**Bancone_et_al_2015 (27)**	Thailand	Purposive, assay evaluation	150	150	Trinity	Sequencing	7.5	34.0	30.6 (18.0 – 58.0)	-
**Bancone_et_al_2016 (28)**	Myanmar	Clinical trial	819	110	Trinity	PCR	7.5	36.5	-	0
**Bancone_et_al_2017 (29)**	Thailand	Routine, health-facility based	13,520	521	WHO (WBC depleted)	PCR	11.8	0.0	26.7 (14.0 – 45.0)	-
**Brito_et_al_2016 (30)**	Brazil	Cross-sectional, assay evaluation	674	101	Pointe Scientific	PCR	6.6	100.0	-	72.0
**Deng_et_al_2017 (31)**	Myanmar	Cross-sectional, prevalence	100	100	Trinity	PCR	5.7	53.0	31.3 (4.0 – 75.0)	0.0
**Johnson_et_al_2009 (32)**	Uganda	Cross-sectional, assay comparison	601	600	Randox	PCR	205[a]	51.8	-	20.0
**Kim_et_al_2011 (33)**	Cambodia	Cross-sectional, assay evaluation	903	155	Trinity	Sequencing	12.6	50.3	25.0 (2.0 – 70.0)	-
**La_Rue_et_al_2014 (34)**	United States	Cross-sectional, assay evaluation	214	110	Trinity	Sequencing	7.2	28.2	33.6 (19.0 – 59.0)	0.0
**Ley_et_al_2021 (35)**	Bangladesh	Clinical trial, cross-sectional and case–control	1680	362	Randox	Sequencing	7.0	48.6	33.5 (6.0 - 80.0	0.0
**Pal_et_al_2021 (36)**	United States	Cross-sectional, assay evaluation	790	35	Pointe Scientific	Sequencing	-	44.4	-	0.0
**Reading_et_al_2016 (37)**	Palestine	Health-facility based	131	79	Randox	PCR	8.7[b]	90.3	2.5 (0.9 – 5.1)	-
**Roca-Feltrer_et_al_2014 (38)**	Cambodia	Cross-sectional, assay evaluation	938	125	Trinity	Sequencing	12	47.2	32.9 (6.0 – 75.0)	-
**Roh_et_al_2016 (39)**	Uganda	Cross-sectional, assay evaluation	631	625	Trinity	PCR	7.9	50.9	2.4 (0.2 – 5.0)	4.0
**Satyagraha_et_al_2015 (40)**	Indonesia	Cross-sectional	2033	70	Trinity	Sequencing	9.9	57.7	33.1 (6 - 70)	0.02
**Satyagraha_et_al_2016 (41)**	Indonesia	Cross-sectional, assay evaluation	610	44	Trinity	PCR+ Sequencing	9.2	54.6	27.8 (6 - 70)	0.02

**Satyagraha_et_al_2021 (42)**	Indonesia	Cross-sectional	1928	367	Trinity	PCR+ Sequencing	11.0	0.0	23.7 (6 - 70)	0.07
**Xia_et_al_2016 (43)**	China	Health-facility based	763	730	Zhongshan Biotech	Multicolor melting curve analysis	1.58[c]	58.4	8.4 (0 - 64)	0.0

*****indicates % of included data from each study, not of entire study sample **;**[a]data only available in units of U/10^{12} RBC, [b]represents mean of 22 G6PD normal males, [c]in units of G6PD/6PGD ratio

Note: '-' indicates data unavailable
