# Supplementary material for: Genetic Variants of Glucose-6-Phosphate Dehydrogenase and Their Associated Enzyme Activity: A Systematic Review and Meta-Analysis
Source: Pathogens. 2022 Sep 14;11(9):1045. doi: 10.3390/pathogens11091045 (PMC9502867; doi:10.3390/pathogens11091045)
Supplement: Supplementary file 1 [file pathogens-11-01045-s001.zip › Supplementary Table S2.pdf]

Table S2: Outliers identified per study

Study	Contributed (n)*	Outliers (n)	p-value**	Number included
**Bancone_et_al_2015 (27)**	101	0	0.09	101
**Bancone_et_al_2016 (28)**	110	4	1	106
**Bancone_et_al_2017 (29)**	276	5	0.13	271
**Brito_et_al_2016 (30)**	2	0	1	2
**Deng_et_al_2017 (31)**	22	14	**<0.001**	8
**Johnson_et_al_2009 (32)**	87	4	0.79	83
**Kim_et_al_2011 (33)**	66	1	0.58	65
**La_Rue_et_al_2014 (34)**	73	0	0.18	73
**Ley_et_al_2021 (35)**	144	7	0.5	137
**Pal_et_al_2021 (36)**	19	0	0.83	19
**Reading_et_al_2016 (37)**	68	2	1	66
**Roca-Feltrer_et_al_2014 (38)**	125	7	0.29	118
**Roh_et_al_2016 (39)**	50	11	**<0.001**	39
**Satyagraha_et_al_2015 (40)**	68	1	0.55	67
**Satyagraha_et_al_2016 (41)**	42	1	1	41
**Satyagraha_et_al_2021 (42)**	243	0	<0.001[a]	243
**Xia_et_al_2016 (43)**	242	4	0.13	238

* excludes wild-type individuals, age <1yr, malaria positive individuals

** p-value from chi-sq test comparing proportion of outliers in each study with proportion of outliers in the rest of the database, including Bonferroni adjustment

[a] % outliers significantly lower than other studies
